# The Effects of 10 Weeks Hangboard Training on Climbing Specific Maximal Strength, Explosive Strength, and Finger Endurance

**DOI:** 10.3389/fspor.2022.888158

**Published:** 2022-04-27

**Authors:** Espen Hermans, Atle H. Saeterbakken, Vegard Vereide, Ivar S. O. Nord, Nicolay Stien, Vidar Andersen

**Affiliations:** Faculty of Education, Arts and Sports, Western Norway University of Applied Sciences, Bergen, Norway

**Keywords:** grip strength, grip endurance, training, sport climbing, rate of force development (RFD)

## Abstract

The aim of this study was to investigate the effects of 10 weeks of hangboard training (HBT) on climbing-specific maximal strength, explosive strength, and muscular endurance. In total, 35 intermediate- to advanced-level climbers (8 women and 27 men) were randomized into a hangboard training group (HBT) or a control group (CON). The HBT program consisted of two sessions of 48 min per week using the Beastmaker 1000 series hangboard, and the following application to smartphone. Both groups continued their normal climbing training routines. Pre- and post-intervention, maximal peak force, maximal average force, and rate of force development (RFD) were measured while performing an isometric pull-up on a 23 mm deep campus rung and jug holds. In addition, finger endurance was measured by performing a sustained dead-hang test on the same rung. The HBT increased peak force and average force in 23 mm rung condition, average force in jug condition, and utilization rate øl,.- in peak force to a greater extent than CON (*p* = 0.001–0.031, ES = 0.29–0.66), whereas no differences were detected between groups in RFD (jug or 23 mm), peak force in jug condition, utilization rate in RFD, average force or in dead-hang duration (*p* = 0.056–0.303). At *post-test*, the HBT group demonstrated 17, 18, 28, 10, 11, and 12% improvement in peak force, average force, RFD in 23 mm rung condition, average force in jug condition, utilization rate in peak force, and dead-hang duration, respectively [*p* = 0.001–0.006, effect size (ES) = 0.73–1.12] whereas no change was observed in CON (*p* = 0.213–0.396). In conclusion, 10 weeks of HBT in addition to regular climbing was highly effective for increasing maximal finger strength compared with continuing regular climbing training for intermediate and advanced climbers.

## Introduction

The popularity of rock climbing is continuing to grow among athletes, recreational climbers, and researchers. The most frequently examined factors in climbing performance are anthropometric-, physiological-, psychological- and technical factors (Watts et al., 1993; Baláš et al., 2012; Saul et al., 2019). Among the physiological determinants of climbing performance, it is generally accepted that both maximal strength and local muscular endurance in the finger flexors are the two most crucial components for predicting climbing performance (Grant et al., 1996; Watts and Jensen, 2003; Watts, 2004; MacLeod et al., 2007; Baláš et al., 2012; Saul et al., 2019), despite a variety of testing protocols (Stien et al., 2022). For example, specific finger strength and dead-hang duration have been found to explain up to 52 and 70% of the total variance of climbing ability (Baláš et al., 2012; Laffaye et al., 2016).

Although finger flexor strength and endurance are the most significant predictors of climbing performance (Cutts and Bollen, 1993; Ferguson and Brown, 1997; Grant et al., 2003; Baláš et al., 2012; Saul et al., 2019), only limited number of finger strength training studies have been conducted (López-Rivera and González-Badillo, 2012, 2019; Medernach et al., 2015b; Levernier and Laffaye, 2019a; Stien et al., 2021a). For example, López-Rivera and González-Badillo (2019) investigated 8 weeks of either maximal hangs, intermittent hangs, or a combination of both in advanced to elite level climbers. All groups demonstrated improvements in finger endurance (i.e., dead-hang duration), but no difference was observed among the three training approaches (López-Rivera and González-Badillo, 2019). Unfortunately, the study did not include any measurement of finger strength or include a control group.

To the best of our knowledge, only three studies have investigated the effects of specific finger strength training and included a control group (Medernach et al., 2015b; Levernier and Laffaye, 2019a; Stien et al., 2021a). Despite similarities, these studies are based on different training protocols, such as dynamic campus board training (Stien et al., 2021a), one arm isometric hangs on the slope and half crimp (Levernier and Laffaye, 2019a), and a combination of different isometric hangs and dynamic exercises (Medernach et al., 2015b). However, none of the three studies demonstrated finger training to be more effective than regular climbing in maximal finger strength measurements. Still, Medernach et al. (2015b), Levernier and Laffaye (2019a), and Stien et al. (2021a) demonstrated improvements in other significant finger training performance outcomes [i.e., finger endurance, campus board moves to failure, and rate of force development (RFD)]. Furthermore, Stien et al. (2021a) examined the upper body strength after the campus board training in a jug and 23 mm rung conditions and demonstrated improvements in isometric pull-up strength in the jug condition, but not in the rung condition (Stien et al., 2021a). Of note, the three studies (Medernach et al., 2015b; Levernier and Laffaye, 2019a; Stien et al., 2021a) included few participants (14–23) on a high climbing performance level (advanced to higher elite) using short intervention periods (4–5 weeks), which may explain the lack of between-group differences in maximal finger strength. Therefore, little is known of the chronic effects (>5 weeks) of finger training on climbing-specific outcomes (i.e., finger strength and endurance) using lower-level climbers (intermediate to advance). The aim of this study was to compare the effects of a longer supplemental finger training protocol while continuing climbing training as usual on climbing specific maximal strength and explosive strength in the finger flexors (primary), and muscular endurance (secondary) in intermediate to advanced recreational climbers. We hypothesized that a specific 10-week hangboard training (HBT) program in addition to regular climbing training would be more effective for increasing specific maximal strength, explosive strength (RFD), and muscular endurance than just continuing regular climbing training.

## Methods

### Study Design

A randomized controlled trial was used to compare the additional effects of combining an HBT program with regular climbing training. After pre-testing, the participants were randomized, by drawing lots, into either the HBT or control (CON) groups. Both groups were encouraged to continue their normal climbing training routine, but the HBT-group completed the hangboard program two times a week. Before and after the intervention, all participants were tested for peak force (F_peak_), average force (F_avg_), and RFD during an isometric pull-up performed on both a 23 mm campus rung and jug holds. Finger endurance was tested during a continuous dead hang test on the same 23 mm campus rung.

### Participants

A sample size calculation was conducted based on the 23 mm rung findings from Stien et al. (2021a). For an alpha level of 0.05 and power of 80%, a sample size of 32 participants appeared to be necessary to detect significant differences between the two groups. There were approximately 70 adult climbers in the climbing club, in addition to 30–40 climbers who were members of the local climbing center who all were asked to participate. To participate, the climbers had to be over 18 years old, free of injuries for the last 6 months, climbing regularly (minimum two times a week), and have a red-point performance level of at least 6a (French grade). Forty-three recreational climbers, who fulfilled the inclusion criteria, volunteered for the study. During the intervention, eight climbers dropped out for various reasons unrelated to the study. Therefore, thirty-five participants (8 women and 27 men) successfully completed the 10-week training intervention. The climbing performance was reported using the French grading system (1-9a/b/c) and converted to the numeric International Rock Climbing Research Association (IRCRA)-scale (1–32) (Draper et al., 2016). Descriptive data for the two groups are shown in Table 1. Before baseline testing, all participants were informed about the study, both orally and in writing, and signed an informed consent form. The study procedures were in accordance with the ethical guidelines of the Western Norway University of Applied Sciences and complied with the standards of treatment of human participants in research, as defined in the 5th Declaration of Helsinki, and were evaluated by the Norwegian Centre for Research Data (2017/56550).

**Table 1:** Descriptive data.

	**CON (*n* = 17)** **Mean ± SD**	**HBT (*n* = 18)** **Mean ± SD**
Age (year)	26.8 ± 7.9	26.2 ± 6.4
Height (cm)	175.1 ± 8.8	175.3 ± 9.2
Weight (kg)	66.7 ± 9.2	70.0 ± 8.7
BMI (kg/m^2^)	21.7 ± 1.8	22.8 ± 2.3
Fat mass (%)	12.4 ± 4.5	13.7 ± 4.5
Climbing experience (year)	7.2 ± 5.8	6.0 ± 6.4
Best red-point (IRCRA)	17.5 ± 4.6	15.5 ± 3.2

*CON, control group; HBT, hangboard training group; BMI, body mass index; IRCRA, International Rock Climbing Research Association*.

### Testing Procedures

All participants were instructed to refrain from any hard physical activity 48 h before testing. Before testing, the participants performed 5 min of an easy climbing traverse followed by 10 min of progressive bouldering (50–80% of their maximum). During the warm-up boulders, the participants had a minimum of 1-min rest between boulders and ~10 min of rest before testing. The test order was standardized and started with three attempts at the isometric pull-up on the 23 mm campus rung, followed by three attempts of isometric pull-up on jug holds. Finally, finger endurance was tested in a sustained dead-hang test on the 23 mm campus rung. Only one attempt was made in the dead hang test.

#### Isometric Pull-Up

**Figure 1:**
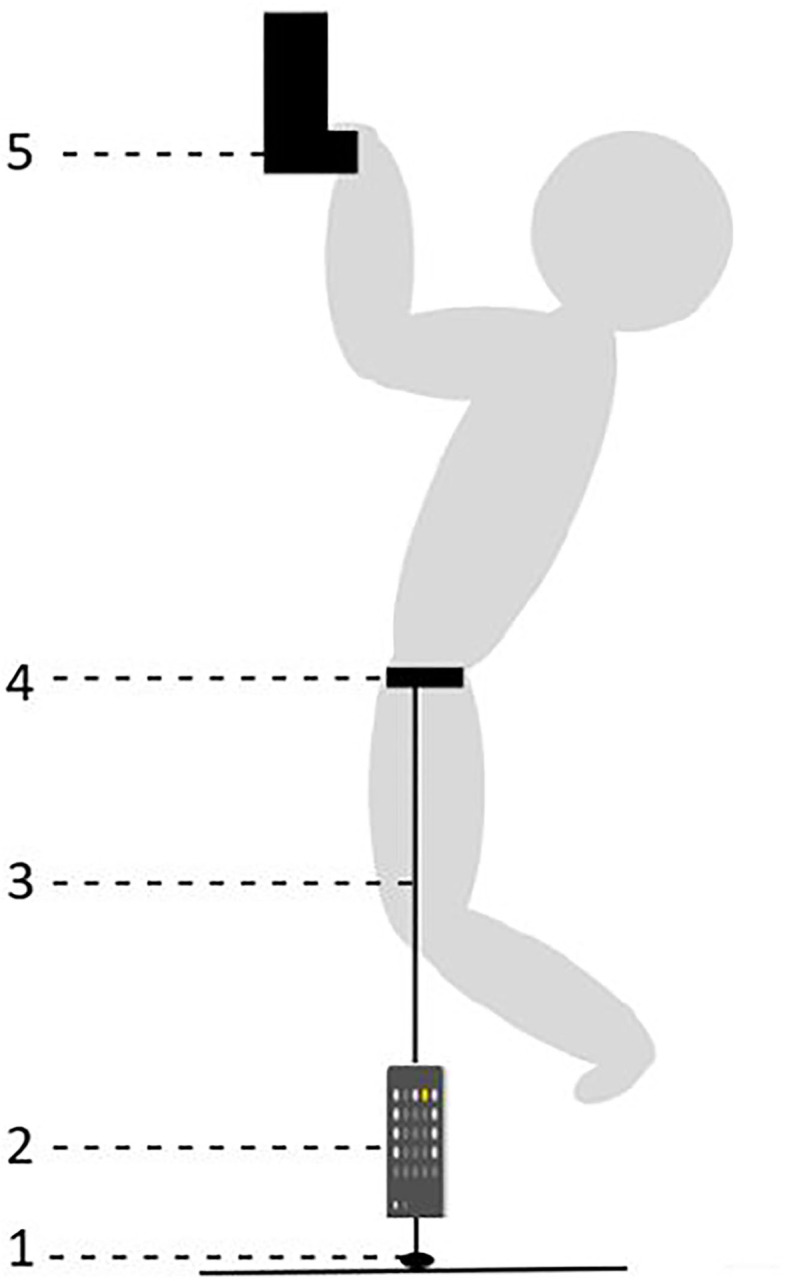
Schematic presentation of the isometric pull-up test showing (1) expansion bolt in the concrete floor, (2) the force cell, (3) the static aramid cord, (4) the climbing harness, and (5) the 23 mm rung or jug holds.

To measure climbing-specific maximal- and explosive strength, an isometric pull-up test was used, ranging from (i) a 23 mm deep and 43 cm wide campus rung with rounded edge (Wood grips Campus Rung size M, Metolius™, USA) and (ii) a wooden jug hold (depth: 30 mm, height: 30 mm, width: 70 mm) on a Beastmaker 1000 series hangboard (Beastmaker Limited, Leicester, United Kingdom). The force (N) was measured with a force cell (Musclelab™ v13.10, 200 Hz, Ergotest Technology AS, Norway) anchored to the concrete floor. The force cell was connected to the climber with a static aramid cord that was fastened to the belay loop of a climbing harness. The climber's position was adjusted (by changing the length of the aramid cord) to 90 degrees angle in the elbow joint (measured with a goniometer) before the test was initiated (Figure 1) (Saeterbakken et al., 2019; Stien et al., 2019). The participants pulled themselves up to the aramid cord that was tight and elbows were in the 90 degrees angle and maintained the position for ~1 s while waiting for the start signal from the test leader. The test leader initiated the test with an oral signal, and the climber performed an isometric pull-up as fast and hard as possible (Maffiuletti et al., 2016), and maintained maximum force for 5 s (Stien et al., 2019). The test was performed with a standardized half-crimp grip with a passive thumb on the 23 mm campus rung, and no restrictions for jug holds. Then, 3 min of rest separated each attempt, and a 5-min rest was given before the jug condition. The attempt with the highest recorded absolute force values for both conditions (rung and jug holds) was used in the analyses. For each isometric pull-up condition, peak force (F_peak_) and average force (F_avg_) were measured in addition to RFD. The F_peak_ was identified as the highest force output on the force-curve and F_avg_ was calculated as the highest mean force over a 2-s period, excluding the peak (Saeterbakken et al., 2019; Stien et al., 2019). For the calculation of the RFD, the change in force output and time window was between the onset of force generation and to the F_peak_, which was found to be the most reliable in the present test (Stien et al., 2021b). The onset of force was detected manually using the commercial software (Musclelab™ v13.10, Ergotest Technology AS, Norway), in line with previous recommendations (Maffiuletti et al., 2016). The relative utilization of force on the 23 mm campus rung relative to the jug holds was calculated as follows; [(23 mm rung results/jug results) × 100] (Stien et al., 2019). Commercial software (Muscellab™ v13.10, Ergotest Technology AS, Norway) was used to analyze the stored data.

#### Dead-Hang Duration

The dead-hang test was performed on the same 23 mm campus rung as the isometric pull-up test after resting for 5 min. The participant was instructed to hang on the campus rung for as long as possible with passive thumbs and fully extended elbows (Watts, 2004; Baláš et al., 2012). The continuous dead-hang test is frequently used and proven to be reliable and valid using a 20–30 mm rung, and is recommended for differentiating climbing performance levels (Baláš et al., 2012; Fanchini et al., 2013; Hermans et al., 2016; Seifert et al., 2017; Stien et al., 2019; Draper et al., 2020). Before starting the test, the participants chalked up their fingers and the campus rung was brushed. To avoid swings, all participants started the test by bending their legs and loading their fingers. Timing started when the participants' legs left the ground and stopped when the feet touched the floor. All participants received motivational feedback and verbal seconding every 10th s during the test. The dead-hang times were measured by the same person using a stopwatch, which has proven to be reliable with an accuracy of 0.1 s (Radner et al., 2017).

### Training Protocol

Prior to each training session, the participants were instructed to perform 15 min of easy climbing or bouldering as a warm-up. The training was conducted on the Beastmaker 1000 series hangboard (Beastmaker Limited, Leicester, United Kingdom) and the participants followed a standardized program using the Beastmaker application (2015, version 3.2.1) on a smartphone. In the Beastmaker application, the difficulty levels (5A−7C) were adjusted based on the size of handholds and the number of included fingers. All training sessions consisted of two identical sets separated by 6 min of rest. Each set includes six progressive hang-series with seven repetitions. One repetition consists of 7 s of hang time and 3 s of rest (7:3 ratio).A 2 min and 30 s rest was given between the different hang-series. To adapt to the training and lower the risk of injuries, all participants were instructed to complete the first 2 weeks on low resistance (5A−5C program). To ensure progression for the last 8 weeks of the training protocol, all participants were instructed to increase the difficulty of the training program when it could be conducted without failure. The program difficulty was increased by changing grip conditions (e.g., smaller holds or fewer fingers) and the order of hold types. The hang time, rest time, and number of repetitions and sets were constant, which made all the sessions last 48 min, regardless of program difficulty. Within the session, 10 min was hanging time on the board. All participants had to fill in a training log where they stated which program they trained for each session. To secure progression in the Beastmaker-program, participants in the HBT group had follow-up sessions on two different occasions with a researcher (week 2–3 and 6–7). The log form also included other types of training, such as climbing, bouldering, and endurance training. This was mandatory for both the training and control groups, to have an overview of total training volume during the intervention program. The participants in the HBT group completed on average ~90% of the training sessions during the 10-week protocol.

### Statistical Analyses

All analyses were performed using the commercial statistical software SPSS (IBM Corp. Released 2020. IBM SPSS Statistics for Windows, Version 27.0. Armonk, NY: IBM Corp). Data were assessed for normality using the Shapiro–Wilk test and all data (*p* = 0.304–963) except dead-hang duration (*p* = 0.010) were normally distributed. Analysis of covariance (ANCOVA) with the pre-test scores as covariates was used to assess differences between the groups for the parametric variables, and independent-samples *t*-tests were used to check for pre-to-post changes within the groups. Differences in training volume were assessed using paired and independent *t*-tests for the within- and between-groups differences, respectively. Dead-hang duration was analyzed using the Wilcoxon signed-rank test for the within-groups comparisons and a Mann–Whitney *U*-test for the between-groups differences. The alpha level was set at <0.05 for statistical significance. All data are presented as mean [± standard deviation (SD)] and Cohen's d effect size (ES). An ES of <0.2 was considered trivial, 0.2–0.5 small, 0.5–0.8 medium, and >0.8 large (Cohen, 1988).

## Results

No differences were observed between groups at baseline for any of the descriptive- (*p* = 0.131–0.960) or performance parameter (*p* = 0.055–0.871).

### Isometric Pull-Up on the 23 mm Campus Rung

**Figure 2:**
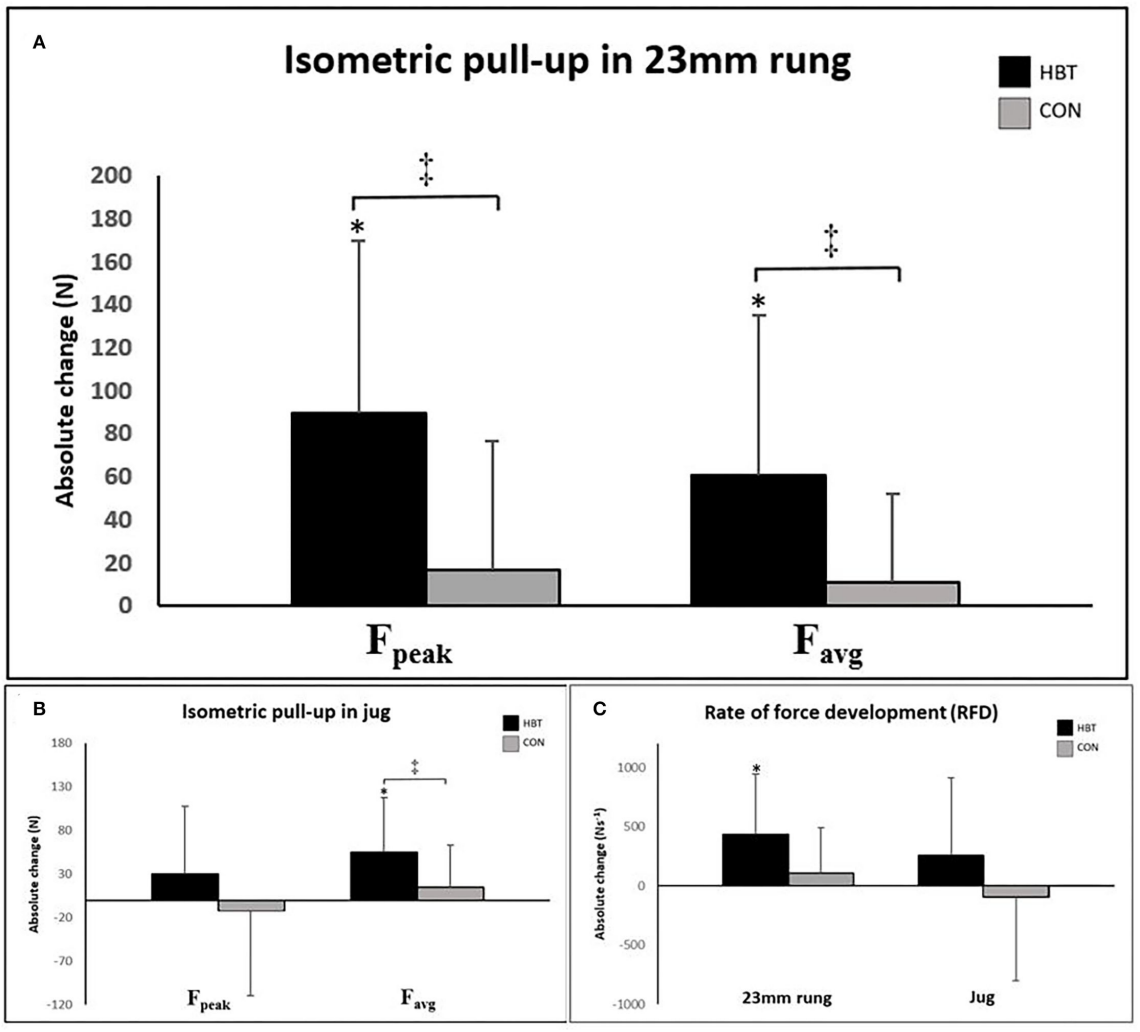
Absolute change in isometric pull-up **(A)** F_peak_ and F_avg_ on the 23 mm campus rung, **(B)** F_peak_ and F_avg_ on the jug, and **(C)** the rate of force development (RFD) in the 23 mm rung and jug conditions. Error bars represent standard deviations (SDs). *Significant change from pre-test (*p* < 0.05). ^‡^Significant difference in change between groups (*p* < 0.05).

The HBT group improved F_peak_ by 89.71N ± 80.25N (*p* < 0.001, ES = 1.12), F_avg_ by 61.19N ± 59.55N (*p* < 0.001, ES = 1.03), and RFD by 436.18Ns-1 ± 506.60Ns-1 (*p* = 0.003, ES = 0.86) from pre- to post-test, whereas none of the parameters increased in the CON group (*p* = 0.213–0.265) (Figure 2). When adjusting for the pre-test results, the HBT group reached a higher F_peak_ (*p* =0.008, ES = 0.31) and F_avg_ (*p* = 0.009, ES = 0.29) at post-test than the CON group, whereas the RFD was not significantly different between the groups (*p* = 0.056, ES = 0.21) (**Table 3**).

### Isometric Pull-Up on Jug Holds

The HBT group improved F_avg_ in the jug condition by 54.94N ± 63.35N (*p* = 0.003, ES = 0.87), whereas F_peak_ (*p* = 0.134) and RFD (*p* = 0.128) (Figure 2) were not increased from pre- to post-test. None of the parameters increased in the CON group (*p* = 0.144–0.871). When adjusting for the pre-test results, the HBT group reached a higher F_avg_ (*p* = 0.031, ES = 0.45) at post-test compared with the CON group, whereas F_peak_ (*p* = 0.143, ES = 0.38) and RFD (*p* = 0.165, ES = 0.23) were not significantly different between the groups (**Table 3**).

### Utilization Rate

The utilization rate of F_peak_ in the rung condition relative to the jug condition significantly improved for the HBT group (10.67 ± 10.54%, *p* < 0.001, ES = 0.73) but not for the CON group (*p* = 0.704). No change occurred in either group for the F_avg_ (*p* = 0.211 and *p* = 0.974). The HBT group increased the utilization of RFD by 9.86 ± 18.51% (*p* = 0.043) whereas the CON group did not (*p* = 0.724). The ANCOVA revealed that the utilization of F_peak_ at post-test was higher for the HBT group than the CON group (*p* < 0.001, ES = 0.66), whereas no between-groups differences were found for the utilization of RFD (*p* = 0.786) and F_avg_ (*p* = 0.593) (**Table 3**).

### Finger Endurance

**Figure 3:**
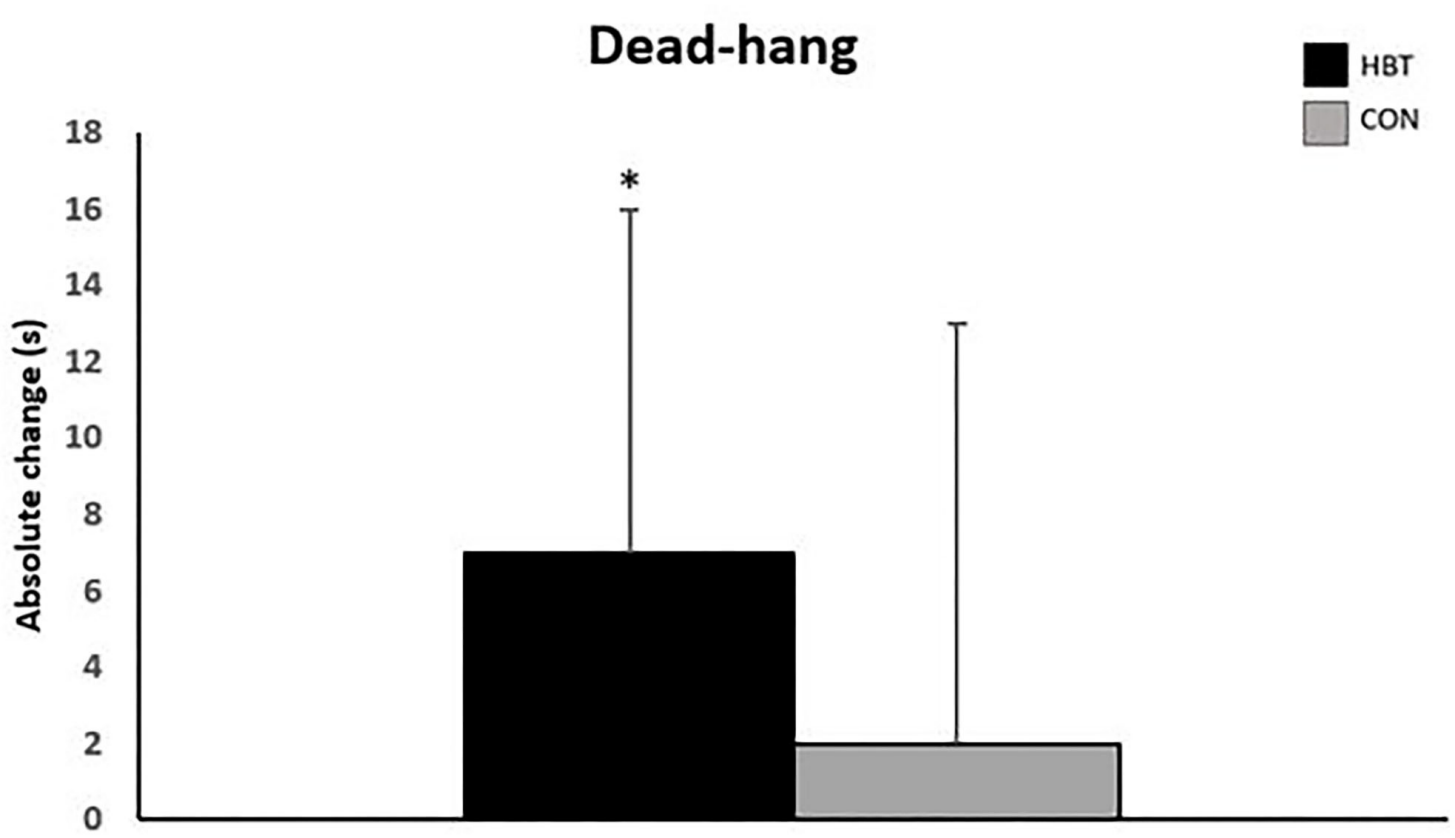
Absolute change (in seconds) for Dead-hang test. *Significant change from pre-test (*p* < 0.05).

A significant improvement in dead-hang duration was observed from pre- to post-test for the HBT group (6.8 ± 8.6 s, *p* = 0.006, ES = 0.79), but not for CON (*p* = 0.215) (Figure 3). The change was not significantly different between the groups (*p* = 0.303) and the groups were not different at post-test (*p* = 0.832) (**Table 3**).

### Training Sessions

A significant difference between groups in the number of climbing training sessions during the intervention was found, with the CON group training more often than the HBT group (*p* < 0.001, ES = 1.00) (Table 2). Of note, for all training sessions (climbing-, strength-, and endurance training), there was no significant difference in the total number of training sessions between the groups (*p* = 0.925).

**Table 2:** Number of self-reported training sessions within the 10-week intervention.

	**CON (*n* = 17)** **Mean ± SD**	**HBT (*n* = 18)** **Mean ± SD**
Climbing and/or bouldering	21.6 ± 6.5	8.8 ± 6.3^≠^
Other training (strength^*^ and endurance)	27.1 ± 12.5	24.0 ± 14.7
Hangboard training program		17.7 ± 1.3
Total number of training sessions	48.7 ± 11.2	48.1 ± 18.5

*CON, Control group; HBT, Hangboard training group*.

≠ *Significantly different from control group, p < 0.001*.

* *Not finger strength training*.

**Table 3:** Absolute data (pre and post) and absolute difference between pre and post for all tests.

			**Hangboard training group**			**Control group**		
			**Pretest**	**Posttest**	**Change**	**Pretest**	**Posttest**	**Change**
Isometric pull-up in 23 mm rung	F_peak_	(N)	425.5 ± 181.5	515.3 ± 167.5^*^	89.7 ± 80.3^≠^	442.2 ± 212.2	462.2 ± 188.0	20.0 ± 73.4
	F_avg_	(N)	282.0 ± 135.3	343.2 ± 149.5^*^	61.2 ± 59.6^≠^	289.8 ± 147.7	302.3 ± 141.2	12.5 ± 41.0
	RFD	(Ns^−1^)	1,098.2 ± 440.2	1,534.4 ± 571.5^*^	436.2 ± 506.6	1,275.1 ± 840.8	1,388.0 ± 829.8	112.9 ± 375.0
Isometric pull-up in jug holds	F_peak_	(N)	662.1 ± 192.9	691.9 ± 172.2	29.8 ± 77.9	616.6 ± 295.6	612.1 ± 251.0	−4.5 ± 115.4
	F_avg_	(N)	501.6 ± 178.7	556.5 ± 172.4^*^	54.9 ± 63.4^≠^	459.2 ± 201.5	476.4 ± 190.0	17.2 ± 47.6
	RFD	(Ns^−1^)	2,138.6 ± 864.3	2,394.6 ± 686.7	256.1 ± 657.8	2,224.2 ± 1,332.6	2,157.9 ± 1,306.1	−66.3 ± 692.2
Utilization rate (23 mm vs. jug)	F_peak_	(%)	63.6 ± 16.8	74.3 ± 13.9^*^	10.7 ± 10.5^≠^	74.9 ± 20.6	77.9 ± 21.2	3.0 ± 18.4
	F_avg_	(%)	57.4 ± 23.4	61.6 ± 17.7	4.2 ± 13.2	62.8 ± 16.8	62.6 ± 14.2	−0.2 ± 11.9
	RFD	(%)	54.0 ± 18.2	63.8 ± 16.2^*^	9.9 ± 18.5	66.9 ± 36.6	70.3 ± 26.2	3.4 ± 35.1
Dead-hang	Duration	(s)	49.4 ± 17.2	56.2 ± 16.8^*^	6.8 ± 8.6	55.8 ± 25.6	58.0 ± 18.5	2.2 ± 11.0

*All results are presented as mean ± SD*.

* *Significantly different from pretest results (p < 0.05)*.

≠ *Significantly different from the control group (p < 0.05)*.

## Discussion

As hypothesized, the present study demonstrated a greater improvement in maximal finger strength (F_peak_ and F_avg_) on the 23 mm campus rung for the HBT group compared with the CON group. In addition, the utilization rate in F_peak_ increased for the HBT group compared with the CON group. Furthermore, 28 and 12% significant improvements in explosive strength and finger endurance, respectively, were observed for the HBT group whereas the CON group demonstrated no improvements between the pre- and post-test. Finally, in contrast to our hypotheses, we found no differences between the groups for utilization rate in F_avg_ and RFD, RFD in jug and rung conditions, F_peak_ in a jug, or in finger endurance.

The HBT program was based on recommendations for resistance training regarding intensity, volume, and frequency (ACSM, 2009). Based on the principle of training specificity and the length of the intervention program, it was not surprising that the finger flexors became stronger which was reflected by the increased performance on the 23 mm rung. Importantly, when performing the isometric pull-up on the jug holds, the back-, shoulder-, and elbow muscles are more important than the finger flexors, as demonstrated by Stien et al. (2019). Hence the training on the hangboard seems to have a small transfer effect on the force-generating capacity of the larger muscles in the upper body, indicated by a positive change in F_avg_ in the jug condition. It could be speculated that the increased performance in F_avg_ is caused by high activation of the back- and shoulder muscles during finger hang training. Of note, the strength tests (rung and jug) were conducted with a 90-degree elbow angle whereas the HBT was conducted with fully extended elbows. Consequently, the improved performance on the 23 mm rung for the HBT group could be a result of better utilization of the force generated rather than an increased force-generating capacity, meaning that the finger strength has developed more than the pulling muscles in the back, shoulders, and elbows. This speculation is supported by greater effect sizes in 23 mm rung vs. jug condition (ES = 1.12, 1.03 vs. ES = 0.87) and the results from utilization rate showing improved utilization for the HBT group in F_peak_.

The fact that the participants in the HBT group improved more than the CON group in all the strength tests performed on the 23 mm rung could also be explained by specificity. The participants in the CON group continued their regular training routine consisting of freely chosen indoor climbing and/or bouldering training, with no training protocols or intensity and volume regulation. Typically, when climbing, all types of handholds are used and the whole body works in varied positions depending on the route, wall angle, and the proportion of support by the legs. In contrast, training on a hangboard is closer to the isometric pull-up testing condition, mainly due to a static activation of fingers and upper body muscles, without the use of the legs. However, unstructured climbing training seems, in this and previous studies, to be an ineffective way to increase the specific strength or climbing performance over a relatively short period of time (Baláš et al., 2012; Medernach et al., 2015a; Hermans et al., 2016; Levernier and Laffaye, 2019a; Stien et al., 2021a). Therefore, it could be speculated that recreational climbing does not provide enough stress or overload to improve the finger strength for intermediate to higher elite level climbers/boulderers in such a short time frame (4–10 weeks).

The present findings, the significant difference between groups in finger strength, are partly in contrast with previous studies examining the effects of finger training (Medernach et al., 2015b; Levernier and Laffaye, 2019a; Stien et al., 2021a). Importantly, all these studies used a short intervention period (i.e., <5 weeks), included climbers with higher climbing ability, and used training exercises that stimulate the larger upper-body muscles in addition to the fingers. However, it could be speculated whether it is the duration of the intervention, the content of the training, the lower skill level of the climbers, or a combination that has led to the difference between the groups in the present study.

In addition to maximal finger strength, RFD is a crucial factor in climbing (Fanchini et al., 2013; Levernier and Laffaye, 2019b). Further, only Levernier and Laffaye (2019a) and Stien et al. (2021a) have investigated changes in RFD after specific finger training protocols in climbers. In contrast to the hypothesis, the present study showed no significant differences in RFD between the HBT and CON groups (28 and 8%, respectively). These findings are in line with Levernier and Laffaye (2019a), who reported a 17% non-significant increase in RFD, using half crimp position following finger training. However, the present study showed a tendency for a difference between the groups for RFD in 23 mm rung condition, which indicates that a longer training intervention could be beneficial in developing RFD. In contrast, both campus training and one-arm isometric hangs are proven to be more effective than climbing/bouldering in improving the average and early phase RFD after just 4–5 weeks of training (Levernier and Laffaye, 2019a; Stien et al., 2021a). However, according to Andersen and Aagaard (2006), the RFD is strongly related to maximum voluntary contraction (MVC) in time intervals later than 90 ms from onset of the contraction. However, no finger strength increase was reported by Levernier and Laffaye (2019a) or Stien et al. (2021a), which indicates that the reported increase in RFD can mainly be explained by neural adaption to the training. Indeed, the findings by Stien et al. (2021b) indicated that the higher RFD observed in elite climbers compared with intermediate and advanced climbers were not caused by the higher maximal strength alone.

The participants in the HBT group demonstrated a 12.1% improvement in finger endurance whereas the participants in the CON group demonstrated no change. Despite this, no differences between the groups were found at post-test meaning that the HBT was not superior to regular climbing training. The test specificity to the training protocol might be an explanation for the lack of difference. For example, the different energy systems contribute differently during intermittent work (e.g., 8:2 and 7:3 ratio) and sustained work (Maciejczyk et al., 2022). Additionally, the intervention was more specific toward muscle strength and less toward muscle endurance. In contrast to the present study, López-Rivera and González-Badillo (2019) reported a 45% increase in hang time after an 8-week intermittent HBT program. Unfortunately, the study had no control group. Of note, López-Rivera and González-Badillo (2019) tested dead-hang duration on an 11 mm campus rung instead of the more common > 20 mm (Draper et al., 2020), which probably present different workload characteristics and are therefore less comparable with the results in the present study.

The present study has some limitations. We found HBT to be effective in improving the performance in climbing-specific finger strength and endurance tests. Although finger strength has been claimed to be important for climbing performance (Grant et al., 1996; Watts, 2004; MacLeod et al., 2007; Baláš et al., 2012; Saul et al., 2019), we cannot conclude that the findings translate to improved climbing or bouldering performance. A climbing or bouldering performance test would have strengthened the study. To test finger flexor endurance in climbers, it is currently recommended to use both an intermittent test and a sustained dead-hang test (Seifert et al., 2017). However, for practical reasons, we could only conduct one endurance test and opted out the intermittent test to avoid favoring the HBT group. It should be mentioned that the intermittent testing (7:3 or 8:2) better reflects the work-relief parameter, with a mean contact time of 8.2 s in sport climbing (Michailov, 2014). However, the dead-hang test is frequently used in climbing research and is proven to be a valid and reliable test with a strong correlation to climbing performance (Baláš et al., 2012; López-Rivera and González-Badillo, 2012, 2019; Medernach et al., 2015b; Hermans et al., 2016; Ozimek et al., 2016; Bergua et al., 2018; Draper et al., 2020).

In conclusion, 10 weeks of supplemental HBT in addition to regular climbing training is more effective for improving the specific finger strength in intermediate to advanced level climbers, compared with continuing regular climbing training. However, the HBT was not superior to regular climbing training in improving RFD or dead-hang duration.

## Perspective

This study aimed to improve the evidence-based knowledge about HBT on climbing-specific strength and endurance tests. The results indicate that a 10-week intervention with specific finger flexor training can be effective to increase a climber's specific maximal strength. Therefore, HBT can prepare a climber for further climbing performance development, using an isometric exercise loaded with only body mass. Stronger fingers can likely allow a climber to hold on to smaller holds and improve the climbing time to exhaustion by letting the climber use a lower percentage of maximal finger strength at a given handhold. These findings contribute to the climbing and research communities' understanding of the effects of a common HBT protocol and should encourage and support both trainers and athletes to include the blocks of supplemental HBT in a periodization program to improve climbing performance.

## Data Availability Statement

The raw data supporting the conclusions of this article will be made available by the authors, without undue reservation.

## Ethics Statement

The studies involving human participants were reviewed and approved by Norwegian Centre for Research Data (2017/56550). The patients/participants provided their written informed consent to participate in this study.

## Author Contributions

EH have written the original draft and EH and NS analyzed the data. EH, IN, VV, and AS completed data collection. All authors have contributed to the conceptualization, critical feedback, editing the draft and have approved the submitted version.

## Funding

This study was conducted without any foundings outside the institution. This study received funds for open access publication fee from Western Norway University of Applied Sciences.

## Conflict of Interest

The authors declare that the research was conducted in the absence of any commercial or financial relationships that could be construed as a potential conflict of interest.

## Publisher's Note

All claims expressed in this article are solely those of the authors and do not necessarily represent those of their affiliated organizations, or those of the publisher, the editors and the reviewers. Any product that may be evaluated in this article, or claim that may be made by its manufacturer, is not guaranteed or endorsed by the publisher.

## References

[B1] ACSM (2009). Progression models in resistance training for healthy adults. Med. Sci. Sports Exercise 41, 21. 10.1249/MSS.0b013e3181915670 19204579

[B2] Andersen L. L. Aagaard P. (2006). Influence of maximal muscle strength and intrinsic muscle contractile properties on contractile rate of force development. Eur. J. Appl. Physiol. 96, 46–52. 10.1007/s00421-005-0070-z 16249918

[B3] Baláš J. Pecha O. Martin A. J. Cochrane D. (2012). Hand–arm strength and endurance as predictors of climbing performance. Eur. J. Sport Sci. 12, 16–25. 10.1080/17461391.2010.546431

[B4] Bergua P. Montero-Marin J. Gomez-Bruton A. Casajús J. A. (2018). Hanging ability in climbing: an approach by finger hangs on adjusted depth edges in advanced and elite sport climbers. Int. J. Perform. Anal. Sport 18, 437–450. 10.1080/24748668.2018.1486115

[B5] Cohen J. (1988). Statistical Power Analysis for the Behavioral Sciences. New York, NY: Lawrence Erlbaum Associates

[B6] Cutts A. Bollen S. R. (1993). Grip strength and endurance in rock climbers. Proc. Inst. Mech. Eng. 207, 87. 10.1243/PIME_PROC_1993_207_275_02 8280318

[B7] Draper N. Giles D. Schöffl V. Fuss F. Watts P. Wolf P.. (2016). Comparative grading scales, statistical analyses, climber descriptors and ability grouping: international rock climbing research association position statement. Sports Technol. 8, 1–7. 10.1080/19346182.2015.1107081

[B8] Draper N. Giles D. Taylor N. Vigouroux L. España-Romero V. Baláš J.. (2020). Performance assessment for rock climbers: the international rock climbing research association sport-specific test battery. Int. J. Sports Physiol. Perform. 16, 1242–1252. 10.1123/ijspp.2020-0672 33652414

[B9] Fanchini G. Violette F. Impellizzeri F. M. Maffiuletti N. A. (2013). Differences in climbing-specific strength between boulder and lead rock climbers. J. Strength Cond. Res. 27, 4. 10.1519/JSC.0b013e3182577026 22505133

[B10] Ferguson R. A. Brown M. D. (1997). Arterial blood pressure and forearm vascular conductance responses to sustained and rhythmic isometric exercise and arterial occlusion in trained rock climbers and untrained sedentary subjects. Eur. J. Appl. Physiol. Occup. Physiol. 76, 174. 10.1007/s004210050231 9272777

[B11] Grant S. Hynes V. Whittaker A. Aitchison T. (1996). Anthropometric, strength, endurance and flexibility characteristics of elite and recreational climbers. J. Sports Sci. 14, 301. 10.1080/02640419608727715 8887209

[B12] Grant S. Shields C. Fitzpatrick V. Ming Loh W. Whitaker A. Watt I.. (2003). Climbing-specific finger endurance: a comparative study of intermediate rock climbers, rowers and aerobically trained individuals. J. Sports Sci. 21, 621. 10.1080/0264041031000101953 12875313

[B13] Hermans E. Andersen V. Saeterbakken A. H. (2016). The effects of high resistance–few repetitions and low resistance–high repetitions resistance training on climbing performance. Eur. J. Sport Sci. 17, 378–385. 10.1080/17461391.2016.1248499 27863457

[B14] Laffaye G. Levernier G. Collin J.-M. (2016). Determinant factors in climbing ability: influence of strength, anthropometry, and neuromuscular fatigue. Scand. J. Med. Sci. Sports 26, 1151–1159. 10.1111/sms.12558 26453999

[B15] Levernier G. Laffaye G. (2019a). Four weeks of finger grip training increases the rate of force development and the maximal force in elite and top world-ranking climbers. J. Strength Cond. Res. 33, 9. 10.1519/JSC.0000000000002230 28945641

[B16] Levernier G. Laffaye G. (2019b). Rate of force development and maximal force: reliability and difference between non-climbers, skilled and international climbers. Sports Biomech. 20, 1–12. 10.1080/14763141.2019.1584236 31038051

[B17] López-Rivera E. González-Badillo J. (2019). Comparison of the effects of three hangboard strength and endurance training programs on grip endurance in sport climbers. J. Hum. Kinet. 66, 183–195. 10.2478/hukin-2018-0057 30988852 PMC6458579

[B18] López-Rivera E. González-Badillo J. J. (2012). The effects of two maximum grip strength training methods using the same effort duration and different edge depth on grip endurance in elite climbers. Sports Technol. 5, 100–110. 10.1080/19346182.2012.716061

[B19] Maciejczyk M. Michailov M. L. Wiecek M. Szymura J. Rokowski R. Szygula Z.. (2022). Climbing-specific exercise tests: energy system contributions and relationships with sport performance. Front. Physiol. 12, 787902. 10.3389/fphys.2021.787902 35140627 PMC8819085

[B20] MacLeod D. Sutherland D. L. Buntin A. Whitaker A. Aitchison I. Bradley J.. (2007). Physiological determinants of climbing-specific finger endurance and sport rock climbing performance. J. Sports Sci. 25, 1433. 10.1080/02640410600944550 17786696

[B21] Maffiuletti N. A. Aagaard P. Blazevich A. J. Folland J. Tillin N. Duchateau J. (2016). Rate of force development: physiological and methodological considerations. Eur. J. Appl. Physiol. 116, 1091–1116. 10.1007/s00421-016-3346-6 26941023 PMC4875063

[B22] Medernach J. P. Kleinöder H. Lötzerich H. H. H. (2015a). Effect of interval bouldering on hanging and climbing time to exhaustion. Sports Technol. 8, 76–82. 10.1080/19346182.2015.1063643

[B23] Medernach J. P. Kleinöder H. Lötzerich H. H. H. (2015b). Fingerboard in competitive bouldering: training effects on grip strength and endurance. J. Strength Cond. Res. 29, 2286–2295. 10.1519/JSC.0000000000000873 26203738

[B24] Michailov M. L. (2014). Workload characteristics, performance limiting factors and methods for strength and endurance training in rock climbing. Med. Sportiva 18, 97–106. 10.5604/17342260.1120661

[B25] Ozimek M. Staszkiewicz R. Rokowski R. Stanula A. (2016). Analysis of tests evaluating sport climbers' strength and isometric endurance. J. Hum. Kinet. 53, 11. 10.1515/hukin-2016-0027 28149428 PMC5260593

[B26] Radner W. Diendorfer G. Kainrath B. Kollmitzer C. (2017). The accuracy of reading speed measurement by stopwatch versus measurement with an automated computer program (rad-rd©). Acta Ophthalmol. 95, 211–216. 10.1111/aos.13201 27572996

[B27] Saeterbakken A. H. Andersen V. Stien N. Pedersen H. Solstad T. E. J. Shaw M. P.. (2019). The effects of acute blood flow restriction on climbing-specific tests. Mov. Sport Sci. 109, 7–14. 10.1051/sm/2020004

[B28] Saul D. Steinmetz G. Lehmann W. Schilling A. (2019). Determinants for success in climbing: a systematic review. J. Exercise Sci. Fitness 17, 91–100. 10.1016/j.jesf.2019.04.002 31193395 PMC6527913

[B29] Seifert L. Wolf P. Schweizer A. (2017). The Science of Climbing and Mountaineering. New York, NY: Routledge.

[B30] Stien N. Pedersen H. Vereide V. A. Saeterbakken A. H. Hermans E. Kalland J.. (2021a). Effects of two vs. four weekly campus board training sessions on bouldering performance and climbing-specific tests in advanced and elite climbers. J. Sports Sci. Med. 20, 438–447. 10.52082/jssm.2021.438 34267583 PMC8256519

[B31] Stien N. Saeterbakken A. H. Andersen V. (2022). Tests and procedures for measuring endurance, strength, and power in climbing – a mini-review. Front. Sports Act. Living 4, 847447. 10.3389/fspor.2022.847447 35308594 PMC8931302

[B32] Stien N. Saeterbakken A. H. Hermans E. Vereide V. A. Olsen E. Andersen V. (2019). Comparison of climbing-specific strength and endurance between lead and boulder climbers. PLoS ONE 14, e0222529. 10.1371/journal.pone.0222529 31536569 PMC6752829

[B33] Stien N. Vereide V. A. Saeterbakken A. H. Hermans E. Shaw M. P. Andersen V. (2021b). Upper body rate of force development and maximal strength discriminates performance levels in sport climbing. PLOS ONE 16, e0249353. 10.1371/journal.pone.0249353 33770128 PMC7997018

[B34] Watts P. B. (2004). Physiology of difficult rock climbing. Eur. J. Appl. Physiol. 91, 361–372. 10.1007/s00421-003-1036-7 14985990

[B35] Watts P. B. Jensen R. L. (2003). Reliability of peak forces during a finger curl motion common in rock climbing. Meas. Phys. Educ. Exerc. Sci. 7, 263–267. 10.1207/S15327841MPEE0704_4

[B36] Watts P. B. Martin D. T. Durtschi S. (1993). Anthropometric profiles of elite male and female sport rock climbers. J. Sports Sci. 11, 113. 10.1080/02640419308729974 8497013

